# Variation and adaptation: learning from success in patient safety-oriented simulation training

**DOI:** 10.1186/s41077-017-0054-1

**Published:** 2017-10-31

**Authors:** Peter Dieckmann, Mary Patterson, Saadi Lahlou, Jessica Mesman, Patrik Nyström, Ralf Krage

**Affiliations:** 1 Copenhagen Academy for Medical Education and Simulation (CAMES), Center for Human Resources, Capital Region of Denmark, Herlev Hospital, 25. Floor, Herlev Ringvej 75, DK-2730 Herlev, Denmark; 2 Children’s National Medical Center, 111 Michigan Ave NW, Washington, DC, 20010 USA; 3 London School of Economics and Political Science, Department of Psychological and Behavioural Science, Houghton Street, London, WC2A 2AE UK; 4 Maastricht University, Faculty of Arts and Social Sciences, Grote Gracht 90-92, Maastricht, The Netherlands; 5 Patient Safety and Learning Centre, ARCADA University of Applied Sciences, Jan-Magnus Janssons plats 1, Helsinki, Finland; 6 ADAM Simulation Center, VU University Medical Center, De Boelelaan, 1117 Amsterdam, The Netherlands

**Keywords:** Simulation, Scenarios, Debriefings, Installation theory, Activity theory, Mundane practice, Patient safety, Safety II, Video reflexivity, Faculty development

## Abstract

Simulation is traditionally used to reduce errors and their negative consequences. But according to modern safety theories, this focus overlooks the learning potential of the positive performance, which is much more common than errors. Therefore, a supplementary approach to simulation is needed to unfold its full potential. In our commentary, we describe the *learning from success* (LFS) approach to simulation and debriefing. Drawing on several theoretical frameworks, we suggest supplementing the widespread deficit-oriented, corrective approach to simulation with an approach that focusses on systematically understanding how good performance is produced in frequent (mundane) simulation scenarios. We advocate to investigate and optimize human activity based on the connected layers of any setting: the embodied competences of the healthcare professionals, the social and organizational rules that guide their actions, and the material aspects of the setting. We discuss implications of these theoretical perspectives for the design and conduct of simulation scenarios, post-simulation debriefings, and faculty development programs.

## Background

During the scenario, the patient developed signs of anaphylaxis. The medical emergency team (MET) was called by the treating nurse. One of the nurses in the MET begins to ventilate the patient. The nurse is tall and reaches easily above the headboard of the bed. Despite effective ventilation, the patient’s saturation is dropping. The MET decides to intubate the patient. The anesthesiologist takes over—she is not as tall as her nurse colleague. The headboard becomes an obstacle. The team struggles a bit to get it loose and out of the way. When attempting to intubate, the light on the laryngoscope malfunctions. The team reverts to bag mask ventilation, while another laryngoscope is fetched. When the patient is intubated, the saturation raises and the scenario unfolds. During the debriefing, the group discusses both situations—the headboard and the broken light. In both cases, the undertone is negative: The laryngoscope should not have been broken, nor should the headboard be an obstacle for the anesthesiologist, as (according to the accepted assumption in the debriefing group) it should have been removed immediately, when it becomes clear that the patient is deteriorating. The team considers options on how to avoid such unwanted variation in the future (learning from failure).

While many debriefing structures emphasize learning from positive as well as negative aspects of a scenario [1–3], the case above illustrates a widespread approach to actual simulation practice as we know it: If there is a suboptimal event during the scenario, the focus during the debriefing is often how it could have been discovered and solved, or better: avoided. Usually, less focus is placed on how participants adapted to the unexpected and recovered the process and their performance to an acceptable level (good performance, or in short: “success”).

In this paper, we discuss how a focus on everyday positive aspects of work can contribute to improving patient safety-oriented simulation. We first explain the theoretical lenses we use as to how learning from good performance makes sense for simulation practice. We then summarize our thoughts and describe implications for the design and conduct of simulation scenarios and debriefings, as well as for faculty development.

## Theoretical perspectives of the learning from success (LFS) approach

In the example above, we could debrief checking routines for equipment that should be standard. This might have helped to identify the broken laryngoscope. We could also debrief standard approaches to treatment including preparation of the work environment. This might have led to the immediate removal of the headboard, when calling the MET. Thus, we could discuss how to avoid errors in the first place and the frequent recommendation to define or refine standard approaches to situations in order to avoid errors. But many such procedures exist already and are not always followed [4]. Adding even more policies and procedures is not necessarily effective. The proliferation of policies can be counterproductive, for example, when procedures and checklists are used in a “tick and flick” approach [5]. The mechanics of checking the boxes may outweigh the thoughtful approach to the task and situation. Our focus here is not on how to avoid errors and unwanted variations but on *learning* how healthcare professionals work towards actively trading-off conflicting goals to find the best possible balance between efficiency, thoroughness, and safety for the individual patient and within the healthcare system. We call this perspective the learning from success (LFS) approach in simulation.

The LFS approach sets the focus on a different angle for the same scenario: How did the team *adapt* to the unanticipated and problematic disruptions? What triggered their adaptations? Why did the actions chosen make sense to those involved? What trade-off considerations were involved? How did the team organize the search for the new laryngoscope while at the same time reverting to bag mask ventilation? When did they become aware of the headboard becoming an obstacle? How did they collaborate to remove it, even though this type of bed was not familiar to them and they thus had to solve a mechanical problem under time pressure? What effect did the time pressure have—on the individuals, on the team, on their treatment of the patient?

The LFS approach focusses on the (re-)creation of good performance based on experience and can help in enlarging the focus of simulation-based training to find positive answers to the many questions in the previous paragraph. Therefore, in simulation, we move the focus away from only concentrating on reducing the occurrence of unwanted deviations and their possibly negative effects—towards reinforcing and increasing efficient and effective adaptation [6–14]. In most cases, our assumption will be that efficient adaptation results in good performance and thus, all other things being equal, better outcome for the patient and team satisfaction. Most of the time, good performance is the result of human adaptation in the face of expected and unexpected perturbations. However, at times, these adaptations seem accidental, as when the participants are not fully aware of how they created the good performance. Focusing on these adaptations is not done automatically, as they are often seen as a “normal” part of everyday work. They are expected and not considered worthy of further thought—as opposed to errors (or conversely brilliant solutions) that trigger attention, arousal, interest, and often strong emotions.

In this paper, we discuss the implications of a new approach for (a) the design and implementation of simulation scenarios, (b) the conduct of debriefings, and (c) the training of faculty. We see our approach as complementary to existing approaches. It is not intended to replace them. Because of its innovative character, we do not have empirical evidence about its effectiveness, but build on the combined experience of the author team and observations in our practices. Our point of departure is to use everyday routine tasks with good outcomes, in a context that brings together people, devices, and rules to understand how healthcare professionals adapt in a goal-oriented fashion to achieve the best outcome for the patient in the given situation. First, we explain our key concepts and their interrelations. Given the number of concepts in this paper, we also provide a summary (see Table 1).

**Table 1:** Key terms and their definition used in this text

Embodied competences	Describes what the person can do without conscious efforts. This can be manual skills; ways of addressing and working with problems (not necessarily solving them); ways of thinking; patterns of interpretations; ingrained assumptions, norms values, and beliefs. They are “they are inscribed in the flesh and emerge as cognitions, emotions and movements.” [32]
Mundane	Describes the regular and yet not trivial aspect of everyday activities. The mundane does not stick out is part of the expectations and routines—and yet, it requires a lot work to keep the mundane and preventing it from becoming extraordinary.
Exnovation	Describes the idea of developing new insights and actions from what is already given. The new ideas are not given “in,” like in *in*novation, but are developed from out of the existing.
Installation	Specific, local, societal settings where humans are expected to behave in a predictable way. Installations consist of a set of components that simultaneously support and socially control individual behavior. The components are distributed over the material environment (affordances), the subject (embodied competences), and the social space (institutions, enacted, and enforced by other subjects). These components assemble at the time and place the activity is performed. [32]

### Opening up the “mundane” as a learning space

Regular everyday practice is valuable for learning on many levels: “Like the mantelpiece clock that nobody notices until it stops ticking, safe clinical practice tends to be taken for granted as a default state. In fact, safety requires an awareness of the complex processes that underlie routine practice, coupled with an ability to recognise problems at an early stage and head them off before they escalate into adverse events. Ensuring that things go right is as important as knowing what to do when they go wrong” [15]. However, concentrating on these mundane activities has challenges and advantages. It is challenging to actually “see” or analyze “normal” events when nothing “remarkable” happens. As these events unfold, they do not necessarily trigger attention and do not invite further thought and cognitive processing. It can be difficult to convince discussion partners to focus on them as relevant, and even if discussed, their underlying dynamics might be difficult to explore: Because of the automaticity of these tasks, they are not easily subject to cognitive analysis.

On the other hand, the mundane is frequent and/or widespread—actions that are done often and/or by many [14]. Thus, mundane actions are valuable in order to understand the corridor of normal performance for an action: What is the variation in task execution over time and/or between different people with the average skill set for the task? Being mindful of these variations can show trends, for example “drifts into failure”—when we are operating in a riskier environment than believed [16, 17]—or emerging good ideas. Any insight, improvement, and learning from such situations would have a large effect, as it would be applied frequently and by many.

Further, these “normal” practices often incorporate a vast amount of experience. The well-executed normal practice contributes to preventing problematic situations and to keeping actions within the expectations, within the corridor of normal performance. Reflecting on the mundane can trigger deep insights about control and prevention strategies and the rationale behind practice that often has been learned without deep reflection. Finally, what is routine for one team may not be routine for another: best (and good) practice can be shared. As a bonus, making explicit success in performance enhances participants self-efficacy (confidence in one’s own ability to achieve intended results), which is likely to affect their future ability to cope with similar situations [18] and in participant’s team spirit.

### A positive perspective enables learning from more than failures

Over the last few decades, a set of theoretical frameworks has evolved that emphasize the value of a positive perspective for learning and design. We describe a number of these to demonstrate some of the conceptual complexity that underlies simulation practice. When looking at the different approaches, their partial overlap and how they supplement each other, the value of interdisciplinary work and research teams become clear. The underlying complexity of simulation requires reflection and is difficult to grasp in streamlined algorithms or primers used to assess simulation practice. Therefore, to make simulation effective, we argue, aspects of these many science traditions are required and need to be used together.

First, one well-established positive approach is *appreciative inquiry*. Appreciative inquiry emphasizes learning from what has worked well in the past and turns these successes into a resource. Moreover, appreciative inquiry considers problems as opportunities or sources of inspiration [19, 20]. The team in the vignette could discuss how their previous experiences helped them in coordinating and what they learnt about their way of coordinating.

*Positive deviance*, on the other hand, has its focus on individuals, teams, or organizations that stand out for excellence. These “positive deviants” should inspire others and spread positive excellent behavior [21, 22]. The team in our vignette could discuss which best practices they had observed during the scenario or other experiences and how they could be replicated.

*Exnovation* aims to explicate the already existing strength of practices in order to improve practices [23, 24]. In doing this, exnovation acknowledges that the “ordinary” is an extraordinary accomplishment and that “things or practices are not less valuable simply because they already exist” [25]. Its point of departure is the idea that mundane and implicit routines of practices have become invisible over time but actually play a crucial role in the foundation and preservation of adequate levels of quality. Therefore, the “hidden” strength of practices should be explicated. With its focus on ways of doing and reasoning that are out of sight, the method of video-reflexive ethnography (VRE) is key to exnovate practices as it provides the required balance between familiarity and unfamiliarity. Known (and filmed) practice can be seen from a new perspective, repeatedly, in slow or fast motion, or other ways that might facilitate new perspectives on the existing processes. In this way, there is a strong overlap between exnovation and simulation in the use of video in a learning space. While watching the video of their scenario, the team may filter out the many adaptation processes they used during the scenario. Many different actions were performed—many of them unconsciously. The video and the exnovation angle take them as something valuable and worth of investigation and reflection.

Finally, in the last few years, we have seen a change of focus in the systems approach in safety research [8]. Whereas *Safety I* has its focus on the avoidance of negative deviations from expected performance, *Safety II* concentrates on systematically understanding how good performance is produced and how adaptive mechanisms help in recognizing perturbations in the system and in reacting appropriately to them. There is a growing recognition of Safety II in healthcare [26, 27] as well as in healthcare simulation [28]. The team in the vignette could discuss and analyze in detail the “normal” and good parts of their performance.

### The context guides human action and experience

The value of an action and its outcome depend on how it fits into its context. Assessing an action or an outcome as positive, as an error, as a brilliant idea etc. is a value statement based on the comparison between what was expected and what was realized in a given context: A task is performed in order to reach a goal [29]. A goal is a conscious representation of the desired result of an activity and at the same time influences which actions must be taken to reach this goal [30]. The actions need to be adapted to the context in which they are taken; successful activity is reaching the goal in the conditions given. Every situation is a new situation, somewhat different from the previous (consider a different patient, different equipment, different colleagues, different mindsets, etc.). Therefore, a good performance in a specific context will inevitably be a subtle adaptation to optimize action in these new conditions. Excellence lies in the capacity to adapt successfully to the dynamic variation of the situation. How can we help the actors seize the complexity of the situation and analyze its components during debrief?

The variability of a situation may be described with three related layers:

1. The embodied competences of the persons involved and the motives that guide their actions
2. The social and institutional rules applicable in the situation
3. The material characteristics of the environment

The embodied competences are all that a human can do, think, experience, feel, etc. They are embedded in the body and are the limits of human performances. Rules can be open or hidden, accepted or opposed, and their official version can be congruent—or not—with their unofficial version. They are also subject to a certain degree of interpretation (for example, the appropriateness of jokes in different settings). Often work as imagined and described in official documents is different from work as done in real work situations [7, 8]. The material layer and what actions it affords (its “affordances”) [31] has obvious influence on what is possible—consider lighting conditions, waiting times for resources, material obstruction, etc. The combination of these three layers is an “installation” that channels (scaffolds and constrains) behavior [32]. Problems may occur when one of the layers is faulty or when they are contradictory. Consider the problem with removing the headboard of the bed. It became a material obstacle, as the rule of removing it was not followed, as the need was not seen previously, since the nurse was tall enough to reach above it. Conversely, one layer can compensate for another. Consider, for example, the collaboration between the team members in removing the headboard, when they find the solution in a shared problem-solving approach (embodied competences). The interplay between the different layers only unfolds when tasks are actually performed: As long as the anesthesiologist does not intubate the patient, the broken light of the laryngoscope is not relevant. The installation as-a-whole produces performance as action unfolds, as in a chemical reaction where the three layers combine.

**Fig. 1:**
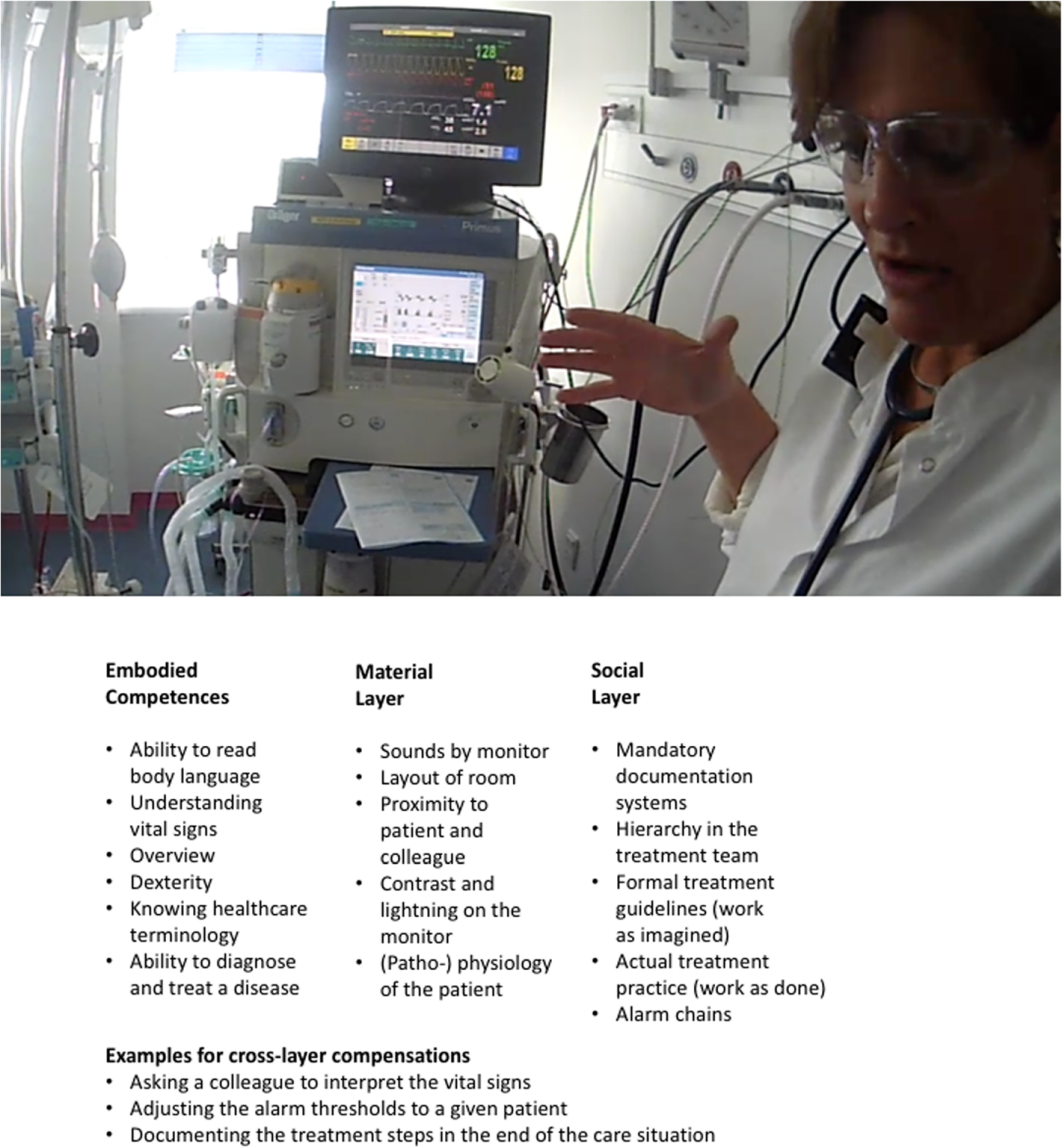
A picture of an anesthesia simulation to illustrate the different layers in an installation with examples

The installation’s three layers provide a framework for enlarging the learning space for the understanding of mundane performance and how it relates to good outcomes. What did the persons involved do, which embodied competences did they draw upon? What guiding rules and standards were there for their actions? What material constraints were relevant (scaffolding or impeding affordances)? Figure 1 illustrates the different layers with a picture from an actual simulation, although not the simulation from the vignette, and examples of elements from each layer. It also provides examples of how the layers can compensate for each other.

We have now described the theoretical perspectives that we would like to use to analyze simulation practice. In addition, we have established the mundane performance as relevant for learning, described different positive perspectives as frames of reference, and explained the standing context layers scaffolding human action. Before turning to the practical implications for simulation practice, we will describe our rationale for why it is important to consider them.

### The relevance of our theoretical perspectives for simulation practice

The real world for which simulation participants train does have variation. The different layers in different contexts will vary, as will their interplay. People are different in their abilities and their performance varies. The material setting in which care unfolds varies, and at times, devices do not function as expected; interpretations of rules and regulations vary—to name just a few examples. Therefore, focussing only on how to avoid variation cannot be a successful strategy to improve safety, as it builds on the flawed basic assumption that variation can be removed from the real world [8]. The ability to adapt to the situation can be seen as one cornerstone of good performance [33–39]. Thus, it is important to include variability in simulation to support its ecological validity—its relevance for the real world [40]. If simulation does not include such variability and does not address helping participants to deal with it, it would prepare participants for an ideal clinical practice, not the real world. Clinicians would be left alone to reconcile what they learned in “ideal simulation” and the “ill-structured” real world of clinical practice [41]. In this sense, participants in simulation should be effectively solving tasks and learn to adapt their actions to achieve their goals within the context variations at the same time [42].

In real life, the competence to flexibly adapt action to diverse contexts is acquired by embodying experiences from repeated practice under varying conditions. Expertise stems from having performed slightly different actions adapted to a variety of slightly different contexts [32, 43]. As a result, the expert, in a given situation, can consider a large array of possible actions in the range of his or her experience, based on analogue situations previously encountered, and can select the most closely adapted. Simulation-based learning can enhance the array of experiences. The learner is not “limited” to experiences from his/her own clinical practice in which some situations might be encountered frequently, while others might be missed altogether. In fact, practicing the seldom, sensitive, critical, and complex situations is a set of key arguments for simulation practice in the first place.

We suggest supplementing such simulation-based learning by using mundane situations in combination with a deeper analysis of what goes well and why it goes well. Simulation offers a systematic way to provide a variety of experiences (within the limitations inherent to simulation). Simulation and the attention to reflection on action also provide the possibility for second-order learning (what *type* of response works), that is the learning of how to produce adapted, non-stereotypic response to a new situation [44–47]. Thus, simulation, and especially the debriefing, offers ways to go beyond the immediately relevant and to analyze underlying connections and dynamics. We believe this is easier in mundane cases than in extreme cases, as the cognitive requirements for processing the mundane typically can be assumed to be lower than what is required for infrequent and complex cases. By analyzing different views and mindsets during the debriefing, new insights about the interplay of the different context layers may be achieved.

Let us give an empirical example. In a debriefing, a very experienced anesthetist watches a video-recording of a difficult airway scenario he was involved in. He explains why he does not comment on a non-critical mistake of the nurse he is working with. His silence was indeed deliberate. His rationale is his belief that if he does rebuke the nurse at that moment, he will jeopardize the full collaboration he needs:

Doctor: “[Not playing the part of] a good team member right now would be very critical. (…) Because we need to be on exactly the same course. We need to stay very good friends right now, because what we’re doing right now is depending completely on each other. And, so opening the risk of any… unwelcome, bad mood is always very risky at this point, and you should never do it (…) because [the nurse] might still act the same, but be angry and then plan to tell me afterwards that she didn’t like something. But then that fills up her brain in some way, and she’ll have to keep room for that, which will give less room for staying in the situation.”

Note that the anesthetist’s primary reason for not discussing the error is *not* to be kind to the nurse. His deep reason is to avoid the nurse wanting to justify herself or talk afterwards for social closure, which he knows will take mental and emotional resources away from the situation at hand. He is not being polite, he is considering the nurse’s cognitive resources to get her full attention and collaboration on the job (and, vice versa, often nurses act the same way when coping with “rude” physicians, for the sake of the physician’s concentration and stress management—such coping mechanisms are not tied to professions). Not saying anything in this situation can be seen as highly efficient communication and a way to collaborate in the moment. The decision to not challenge the nurse could, in psychological terms, be seen as a strategy to avoid tension from an “open issue”—a well-known, resource-intense tension known as the Zeigarnik effect [48]. This draws attention to a generic positive competence of considering the collaboration with others in the light of mundane actions. The dynamic underlying example is seen often and in many domains—it can be considered mundane: it is not a matter of doctors vs. nurses, male vs. female, novice vs. experience. It can happen in any care settings, and it will always pose challenges for effective communication in the light of conflict or just different thoughts about how to proceed. By combining the phenomena observed with theoretical perspectives and models from various disciplines (including psychology, sociology, and anthropology to name a few), work as done in practice and with its variability can be understood in more detail.

The LFS approach has implications for simulation practice. In practice, we advocate for a combination of both the positive and the deficit approach for scenario design and implementation as they can complement each other. Simulation should address the rare, critical, and sensitive situations, as well as the mundane and frequent situations.

### Summary of the foundations of the LFS approach

**Fig. 2:**
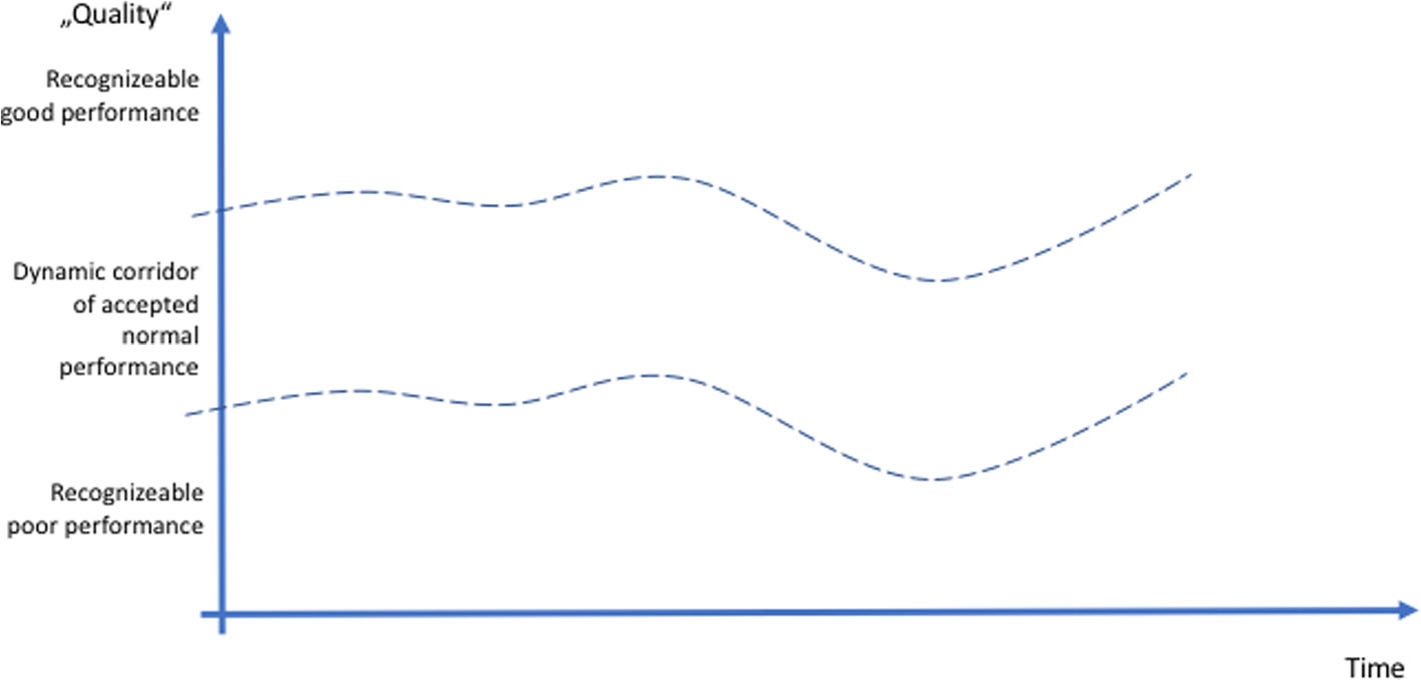
The dynamic of the moving corridor of normal performance, as defined by individually or professionally accepted good practice

**Fig. 3:**
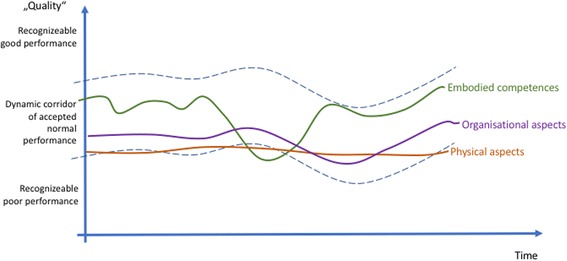
There are constant variations on the three levels of the installation: embodied competences, social- and organizational rules, and the material layer

**Fig. 4:**
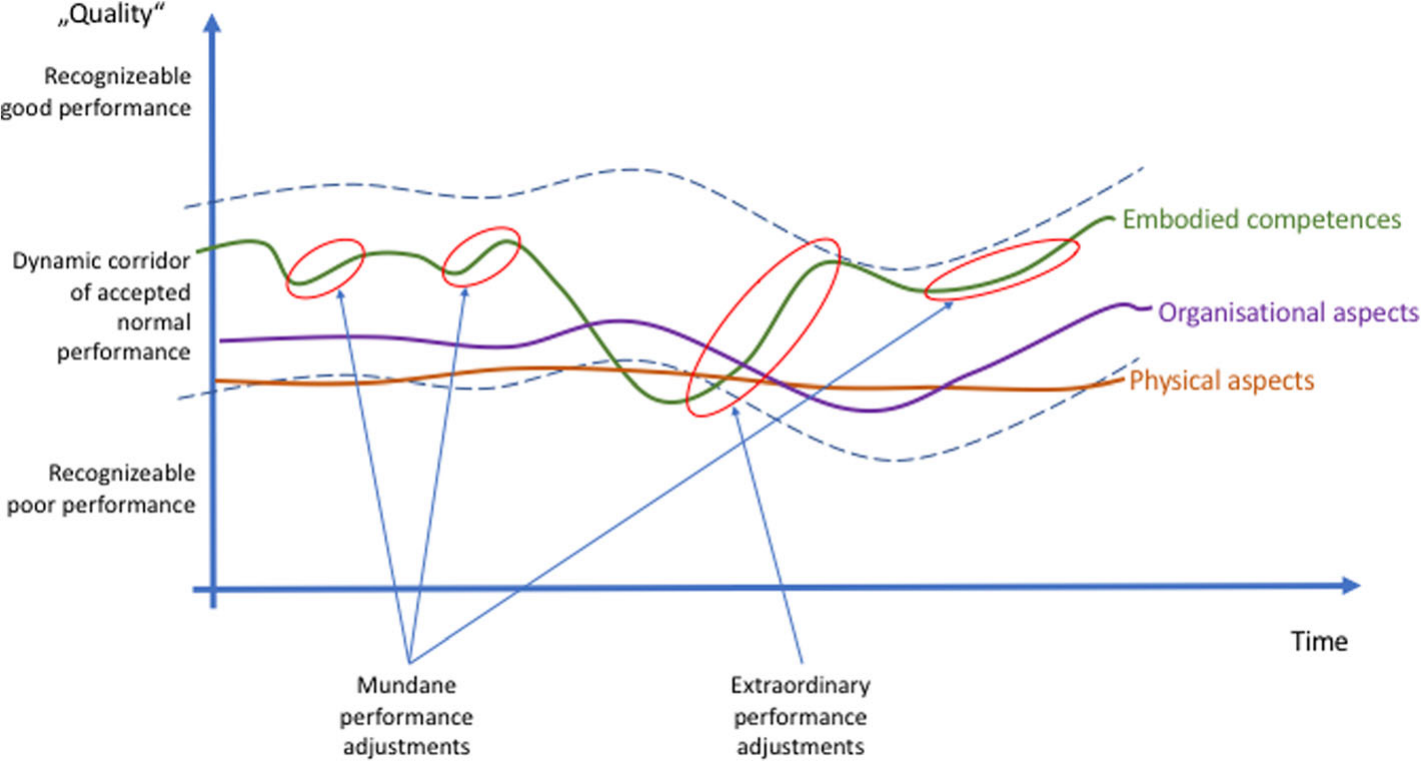
Different possible focus points during debriefings

**Fig. 5:**
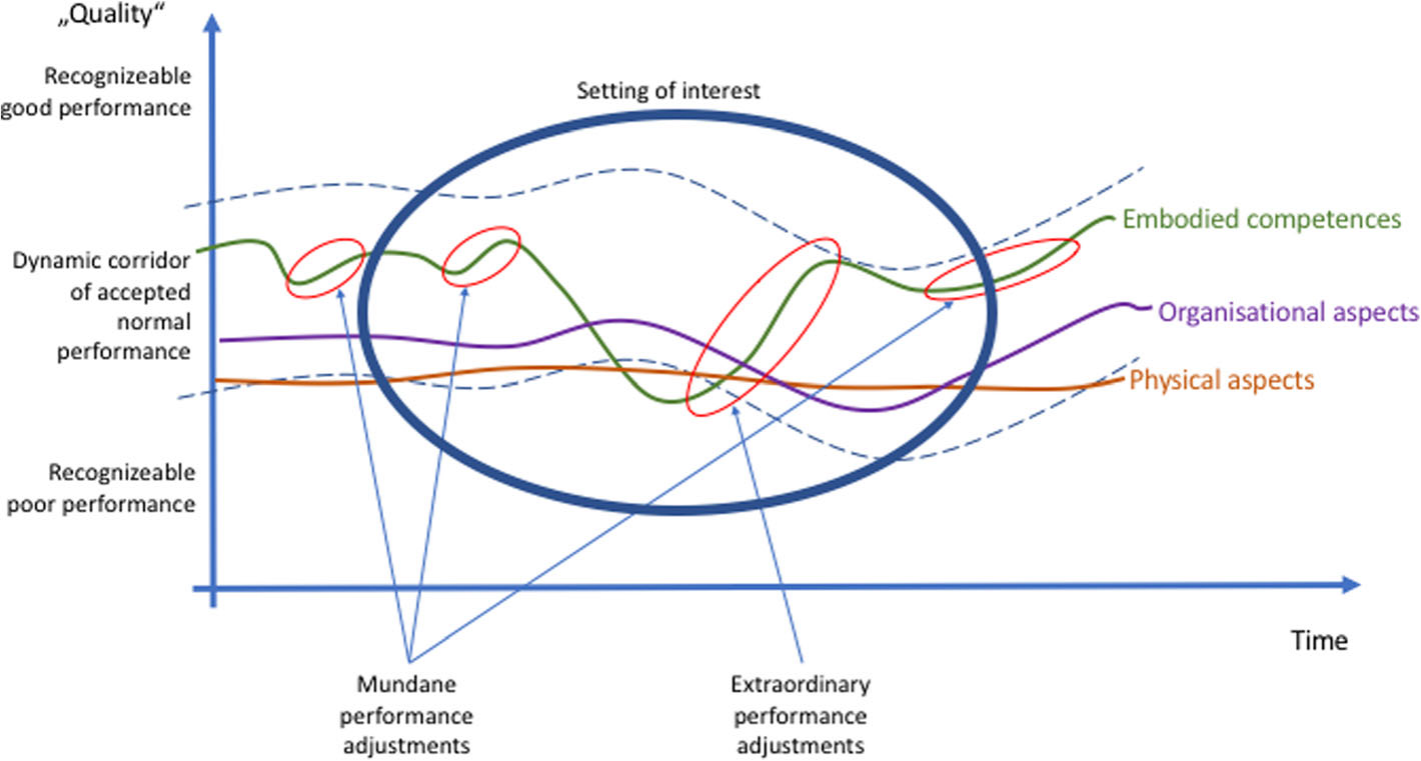
Limits in time and space influence, which aspects of the scenario are discussed

Figures 2, 3, 4, and 5 provide a summary of our considerations that are the base for the practical implications we draw for the design and conduct of simulation-based training.

For each process, there is a subjective and/or objective measurement of its quality. Some criteria are measurable (although the results of the measurement still require human interpretation), while others are based on subjective assessment. The profession, a single person, a group of people will consider certain performance as “normal,” other performance as especially good, still other performance as poor. This corridor of normal performance is dynamic. What is considered “good” might be considered poor practice tomorrow or in 50 years [49]. Different people, professions, disciplines, or cultures might define the corridor of normal performance differently, based on variations in norms, values, and beliefs. Therefore, the corridor of normal performance is depicted in a curved fashion (Fig. 2).

All the layers in the installation show variability: human action varies, organizational procedures change. Even machines and devices function with variability—consider break-downs for example. A lot of the variation stays within the corridor of normal performance. Some, however, is recognized as good or poor. It will be more easily recognized the more the variation goes above or below the corridor of normal performance (Fig. 3).

Traditional simulation practices often focus on the negative, especially in human performance. The larger and steeper the gap in performance, the higher the likelihood that an issue is addressed. This is especially likely when the border of the corridor of normal performance is crossed [45]. Some debriefing structures suggest to also look at positive movements [1, 2], but the implementation of this intention does not seem to function fully in practice. We emphasize in addition to not only look at the extraordinary positive adjustments, but also at the mundane adjustments. The mundane, small adjustments occurring *constantly build*, *rebuild*, *and transmit the foundation of good overall performance* (Fig. 4).

Each simulation-based course or clinical debriefing is a snapshot of the overall system. The setting under consideration is limited in time and space. Events before or after and events outside of the scope of the investigation might never become a consideration for the current discussion, even though they might explain the history of the dynamics in the installation and help to anticipate future developments (Fig. 5).

## Implications for scenario design

As noted for debriefing, some of the existing frameworks prescribe similar approaches as our paper—this is also true for the design and implementation of scenarios [50–54]. Therefore, some of the suggestions may seem familiar. Yet, simulation in theory does not always translate to simulation in practice. In addition, we relate frameworks with each other that in a new combination to allow us to approach the complexity of simulation as social practice [55]. Thus, we base our approach on exnovation principles: we look at existing approaches and practice and develop new thoughts from this.

The LFS approach provides a structure for systematic investigation of actual factors and components used in pursuing goals. All three context layers (embodied competences, social and organizational rules, and material aspects) can be used to systematically investigate and design scenarios that maximize the experience range offered to participants. The material layer may or may not provide what is needed for the task. The people involved could have the right skills for the situation, they could also be under- or overqualified. The rules that guide the task execution might be known and accepted by all involved, they might also be unknown or opposed by some of the participants in a scenario. Systematically varying the layers and their interplay allows exploration of how a work system functions in more detail. For example, what happens to a scenario when a less experienced person participates? Such systematic variation can be used to analyze why the system is functioning well: how was the inexperience of the novice compensated for? Is the integration functioning well and how could it be improved even further? What are working strategies of novices and what of experts? What different errors would they be involved in? Designing scenarios based on the insight into the dynamics of its components may be seen as a strong test for the analysis of the system’s effectiveness. If we understand how good performance is produced, then we should be able to design scenarios in which participants systematically demonstrate good performance and are still able to adapt to the changes introduced. This approach of enabling or supporting good performance can inform scenarios that are designed to help participants improve their performance. Hollnagel’s *Functional Resonance Analysis Method* provides another approach on the systematic variation of different aspects of scenarios [7]. Such systematic variation could be combined with existing scenario design methods [56–61].

In practical terms, this would suggest the need to design more scenarios around mundane situations of care [15]. These situations could then be simulated with a higher degree of ecological validity by capturing the variability typical for the actual clinical situation across the context layers. Such systemic variation could enable the identification of the key elements that make a difference in reliably creating good performance. This knowledge could be incorporated during debriefs.

Another way to build scenarios on the LFS approach would be to include all the stakeholders in the scenarios that would typically be involved in clinical care. In current practice, some of the positions are often introduced as role players, instead of the actual professionals—often for financial and logistic reasons. In inter-professional, inter-departmental, inter-disciplinary scenarios that focus on the mundane collaboration of the stakeholders around patients, it is important to stimulate learning situations for all involved. In the LFS approach, there might be potentially more stakeholders involved—also those on the other side of the hand-over (e.g., the personnel in the recovery room for anesthesia scenarios) or patients, as, after all, they and their values are in the focus of simulation activities [62–64]. Of course, resources needed should be in balance with learning goals and the overall system in which they are used.

For the implementation of such scenarios, it would be helpful to anticipate possible cues that would enable the scenario to play out within the mundane areas, not drifting (too much) outside of the corridor of normal performance [56]. Because of the variation in context layers, even “simple” scenarios might easily develop a complexity that is not anticipated—especially, if the discussions in the debriefing reach deeper reflections.

The more information such simulations of mundane situations demonstrate concerning the normal functioning of the healthcare system, the more this knowledge could be used to design scenarios that are even more relevant to clinicians. In this way, a stronger feedback loop between training results and training design can be established. Triggers for deviations, error-traps, known good solutions, etc. could be used to design scenarios that systematically help create a learning opportunity for participants:

- For example, a sequence of scenarios could show the different aspects of handing-over the patient between different departments in a hospital. The difference of the LFS approach to some of the existing approaches lies in the focus on the mundane hand-overs, not the difficult ones. By involving the different stakeholders in a participatory approach to the design and conduct of such training and by jointly discussing the scenarios, connections could be made and mutual understanding could be increased. Here, in situ simulations might be beneficial [65–69].
- Good solutions for handling a certain patient could be distilled from various teams in simulations. Such a model of good solution could then be introduced to other teams to explore, what kind of adaptations the different team composition and a change of context would require.

## Implications for the debriefing

The LFS approach requires efforts to help participants see the benefit of analyzing good performance in mundane situations. Many participants expect challenging scenarios that stretch them to the limits and debriefings in which each error is addressed and corrected. They do not expect to analyze “the regular stuff” and why it turned out well, nor do the learners necessarily value or even recognize the variation that occurs as part of everyday work. With a focus on good performance, participants are potentially more open for a detailed reflexive discussion about the internal processes that guided their actions. With this openness, it is easier to understand differences in the individual frames held by different persons about a certain situation. Understanding those frames in detail requires an open exchange, not hindered by defensive behavior, which is more likely to occur when focusing on errors [42, 55, 70–75]. Research shows that in-depth analysis of good performance in the context of a study provides access to almost the same learning points as the analysis of mistakes [76]. Such a detailed analysis would help in the previous vignette, for example, to understand the triggers to begin coordinating the search for another laryngoscope; the verbal and non-verbal agreements made between the team members to coordinate their actions; the monitoring of the progress in the search; the trigger to remove the headboard from the bed; and the coordination of actions to jointly find the solution to remove it. In a next step, this reflexive discussion could center on ways of applying similar principles in different situations.

While many published debriefing structures contain the analysis of positive points, in practice, there seems to be a marked difference in the conversation around errors and positive performance. Errors are analyzed in some depth; good performance aspects, however, are often mentioned only superficially. Discussions of good performance should address all the context layers described above: What are the resources and conditions that enhance and enable the team’s ability to adapt? This is not an easy task and requires the facilitator to stimulate deep reflections about what is often taken for granted [77]. Simulation debriefings offer many of the elements that make this discussion relevant: a trained facilitator, to help participants to relate their actions to safety and human-factors theories in addition to the clinical aspects; video recordings offer an outside view; protected time and space to engage in meaningful discussions; and (hopefully) a working agreement (ground rules) that enables the necessary trust for a discussion that analyses the “taken-for-granted” [24].

In order to create the atmosphere needed for such an open exchange, many measures to ensure the psychological safety of those involved are important [42, 74, 75, 78–80]. One is the clear distinction between descriptions and interpretations of the underlying perceptions.

Research shows that oriented, in-depth analysis of successful “normal” situation handling can provide access to almost the same learning that is generated from “mistakes,” provided the discussion is properly oriented. Lahlou and his working group use videos, filmed in a first-person perspective (Fig. 1 provides a still from such recording) and ask participants to comment on their own videos in so called replay-interviews. The study team systematically asks participants not only about their positive goals (“what state of things were you trying to attain?”) but also about their negative goals (“is there anything you were trying to *avoid happening* in acting so?”). This procedure makes explicit the potential issues and risks that were at stake for the clinician, and highlights positive and dynamic safety aspects. This also often triggers narratives of variants of the filmed situations and gives an impression about the realistic variability of work as done. Mesman and colleagues have also successfully used video reflexivity in the Mayo Clinic, USA, while applying the positive approach and focusing on very mundane day-to-day activities in order to analyze inter-professional collaboration between breast cancer surgeons and pathologists. While watching the footage of their own practice, each team realized just how complex was the work of the other team and of themselves. Surgeon: “So one, I’d say my biggest impression from looking at this video is how complex it really is, and we do this every day and we just take it for granted.” They also learned how much they actually do just to facilitate the work of the other team. This resulted in re-appreciation and re-awareness of both the work of the others as well as their own. Crucial in these reflexive meetings was the positive approach.

Also, when using the LFS approach, it is common to observe errors in the scenario. Some might be small and in the light of limited debriefing time might not be discussed, as other angles might be prioritized. Other errors, on the other hand, might be serious enough to warrant a discussion. In some cases, it might not be possible to discuss all errors. Each case will need a judgment call by the debriefer. We see a goal for debriefing that all relevant errors are mentioned and that this is agreed upon with the participants during briefing. If warranted, time might have to be assigned for this discussion within or even after the debriefing—a challenge of adapting debriefing practice to the situation. Another practical approach could be to analyze how the team prevented negative consequences from the error, mitigating possible damage. Alternatively, the participants may be asked to reflect good performance. Again, we emphasize that the LFS approach would be used in combination with debriefings that focus on errors in more detail. Not every detail can be discussed in a session; the goal of training is to learn something important, not to discuss everything. The desire for exhaustive analysis should not get in the way of a deep discussion of one specific aspect that takes time but leads to an important generic insight.

In practical terms, debriefings in the LFS approach might benefit from:

- Seeing selected relevant aspects of the scenario recording more than once, as the focus of participants is often on the negative aspects, when they see the video for the first time. Various recording systems allow for viewing of discrete sections marked by the facilitator rather than the entire video.
- Consciously observing scenarios and collecting material for the debriefing that focus on adaptations and success.
- Looking systematically at the influence of the three layers of the installation and not only at the embodied competences to enhance a systemic understanding of situations.

## Implications for simulation trainers and faculty development

Faculty development is under much debate in healthcare simulation, as well as in medical education [70, 81–88]. Classically, a key ability of faculty for implementing the positive approach is listening to what was said and observing what was done. Faculty need “passivity competence” [24], meaning the ability and willingness to first observe and listen instead of acting immediately. This delayed response enables participants “to become sensitized to a greater array of impressions” [24] which expands their capacity for effective action. It moves the focus to what learners actually say and do. This requires so-called collaborative attention, the capacity of “having the patience to be open—open to what colleagues are doing and saying, open to the implications of what they are doing and saying, and open to colleagues having questions about what is appropriate to do and say” [24]. Workshops and courses or exercises for interviews often show how difficult it actually is to really listen to another person and to perform appreciative inquiry. Thoughts about the next question, early interpretations, stereotypes, and many more aspects influence how much (or little) faculty actually understand concerning what participants are saying. Slowing down, concentrating on core issues, and trying to ensure accurate understanding of what the other person said should be a core competence. This also includes becoming aware of each participant’s contribution to the conversation in terms of motivations, feelings, assumptions, etc. Following Schein’s “humble inquiry” recommendations may be useful [89].

For the LFS approach, many of the abilities faculty need are similar or identical to those needed for faculty in general simulation practice [70, 90]. Our approach, however, would require an improved understanding of core concepts around human action that unfolds in contexts. To guide reflections around these issues likely requires a more in depth understanding of the science behind human factors, patient safety, implementation science, and organizational psychology, to name a few. It is about becoming confident using a new vocabulary and new concepts. In practice, this could be initiated by systematic, check-list like, investigations of the three context layers during a debriefing. The aim is to stimulate participants to make explicit the material scaffoldings, the skills, and the rules that were used in producing good practice. Deep understanding of the concepts likely would require substantial study and experience.

Experience from discussing the LFS approach with faculty shows that there are several concerns raised. Some issues relate to the interaction with low performing learners, the need to correct errors that did occur during the scenario, and dealing with mutual expectations. In many countries, the traditional role of the teacher includes identifying and correcting errors via clear feedback of what is right and wrong especially with low performing participants [33, 71, 91]. Debriefers can easily become the most active members during such discussions, focussing on teaching and not on questioning, reflecting, and understanding [91]. Novice faculty members need to understand the dynamics of such role expectations and reflect upon how much they should concur with or violate them in order to optimize the learning opportunities for the various learner groups in the different work settings.

The approach we suggest here requires sufficient time to discuss the activities and thoughts of the participants during the scenario. Studies show that current debriefing practice may not reach this deep reflective level consistently; too little time for a large number of topics might be one of the obstacles for deep reflections [77]. It may be enough to focus on a few key moments of the scenario to create the habit and reflexive mindset of learning from success, which participants can then carry into their daily practice [44]. Applying the in-depth analysis of the LFS approach, participants will likely obtain insights that they can generalize. In this perspective, simulation debriefing also has the higher-order learning objective to train participants to be analytic and reflexive on their actions on an everyday basis. Table 2 summarizes our practical considerations and contrasts the LFS approach with traditional simulation practice. In practice, both approaches will likely combine and overlap.

**Table 2:** Comparison of traditional simulation-based education with the LFS approach, based on selected phases of the simulation setting [72]

Simulation setting phases	Traditional approach	Learning from good performance approach
Pre-briefing and setting introduction	Emphasis on the extra-ordinary and possibility to train rare, critical, sensitive, and complex situations. The debriefer as (facilitating) expert.	Emphasis on the value of existing mundane practice. The debriefer as partner in the common learning process.
Scenario Conduct	Aim to find the edges of the participants’ competences. Generation of stressful conditions. Use of error traps to generate debriefing-related experiences. Complex scenarios in regards to clinical care, human factors issues, and the use of the simulation.	Aim to work through common scenarios including systematic variation along the FRAM [7] aspects: • Trigger for an action • Outcome of an action • Prerequisites for an action • Resources needed while action is performed • Time aspects • Control mechanisms and rules for the action
Debriefing	Focus on failure and how to avoid them. Positive performance mentioned and praised, but not analyzed. Focus on events “sticking out”: gaps and peaks—failures and good ideas.	Focus on how to systematically produce good performance by adjusting team and care processes to the context. Focus on the deep analysis of good performance and how to reproduce and re-apply it. Focus on performance within the corridor of normal performance.

The table emphasizes the contrasts. In practice, both approaches will overlap considerably and/or supplement each other

## Future directions

Using exnovation principles, we analyzed existing simulation practice and reflected how it could be supplemented with the LFS approach. Our approach brings together a substantial number of frameworks. All align in terms of understanding how positive adaptation was reached rather than only focussing on failures. They provide a grid for analysis as well as some practical techniques that colleagues may use in practice. Comparative studies will be needed to identify which topics are actually discussed in LFS-oriented scenarios and debriefings. It will also be important to understand what effects this has on the participants’ reactions, learning, and its applications in clinical settings. Ultimately, we need to understand how this application influences patients’ experiences and outcomes. Such studies will enable us to discover the best types of situations to use LFS in simulation training. We anticipate some effects will be difficult to capture with traditional study designs, because of the complex situations that the LFS approach addresses. Therefore, we believe that a focus on the processes that are enabled in the LFS approach—understanding the system and performance interactions that enhance good performance—would be a good place to start to create a reflexive culture of LFS.

## Conclusion

We described an innovative approach to simulation and debriefing—the LFS approach. LFS focusses on the systematic understanding of how humans with their embodied competences act in the context of social and organizational rules and material context to create good performance in mundane situations. To supplement traditional simulation approaches with this perspective, scenarios should be based on common, everyday situations, and debriefings should focus on a detailed analysis of how good performance was produced. Simulation faculty can use theoretical insights, terminology, and practice described in this paper to implement this approach.

## Abbreviation

LFS: Learning from success
